# Emerging technologies in thoracic surgery: artificial intelligence, robotic-assisted surgery, and telesurgery

**DOI:** 10.3389/fsurg.2026.1890003

**Published:** 2026-08-18

**Authors:** Zaid Yacoub, Mohammad Shweiki, Rita Yacoub, Nabil Khan, Hamzeh Yacoub, Mohammad Eid Al Mohtasib, Anas Salhab, Fahim Kanani, Firas Abu Akar

**Affiliations:** 1 Faculty of Medicine, Al-Quds University, East Jerusalem, Palestine; 2 Department of Thoracic Surgery, Thoraxklinik, University of Heidelberg, Heidelberg, Germany; 3 Translational Lung Research Center Heidelberg (TLRC-H), German Center for Lung Research (DZL), Heidelberg, Germany; 4 Faculty of Medicine, Arab American University, Ramallah, Palestine; 5 Department of Thoracic Surgery, The Edith Wolfson Medical Center, Holon, Israel; 6 Gray Faculty of Medicine and Health Sciences, Tel Aviv University, Tel Aviv, Israel; 7 Department of General Surgery, Faculty of Medicine, Al-Quds University, East Jerusalem, Palestine

**Keywords:** artificial intelligence, radiomics, robotic-assisted thoracic surgery (RATS), telesurgery, thoracic surgery

## Abstract

**Background:**

Emerging technologies are increasingly reshaping thoracic surgery across the continuum of care, from imaging-based diagnosis and risk stratification to operative planning, minimally invasive intervention, postoperative monitoring, and remote surgical delivery. Artificial intelligence, robotic-assisted thoracic surgery, and telesurgery represent three major domains with the potential to improve precision, personalization, and access to specialized thoracic care.

**Objective:**

This review aims to synthesize current evidence on the applications, clinical value, limitations, and future directions of artificial intelligence, robotic-assisted surgery, and telesurgery in thoracic surgical practice.

**Methods:**

A narrative review of recent literature was conducted, focusing on studies and reviews addressing artificial intelligence–enabled thoracic imaging, pulmonary nodule assessment, radiomics, three-dimensional reconstruction, intraoperative navigation, perioperative risk prediction, robotic thoracic procedures, and 5G-enabled telesurgical systems.

**Findings:**

Artificial intelligence has demonstrated increasing utility in pulmonary nodule detection, malignancy risk estimation, automated segmentation, surgical planning, augmented intraoperative visualization, and prediction of postoperative complications. Robotic-assisted thoracic surgery has expanded the scope of minimally invasive thoracic surgery by providing enhanced three-dimensional visualization, articulated instrumentation, improved dexterity, and favorable perioperative outcomes across lung, mediastinal, and esophageal procedures. Telesurgery, particularly when supported by ultra-low-latency 5G connectivity, has shown early feasibility in extending expert surgical care across long distances and enabling remote collaboration.

**Conclusion:**

Artificial intelligence, robotic-assisted surgery, and telesurgery are redefining thoracic surgery toward a more precise, data-driven, minimally invasive, and globally connected discipline. Responsible integration will be essential to ensure that technological innovation translates into safer procedures, improved outcomes, and equitable access to advanced thoracic surgical care.

## 1 Artificial intelligence in radiology, diagnosis, and surgical planning for thoracic surgery

### 1.1 Introduction

Recent advances in artificial intelligence (AI) have reshaped multiple phases of thoracic surgical care. No longer limited to experimental applications, AI now plays a pivotal role across the continuum of practice, with the potential to transform the future of thoracic surgery. Its uses extend from radiologic diagnosis and malignancy risk stratification to preoperative planning, intraoperative navigation, and postoperative management (1, 2). Throughout this section we distinguish applications that operate primarily within the radiology and pulmonology diagnostic workflow—nodule detection, characterization, and malignancy risk stratification—from those that act directly on the surgical episode, namely operative planning, intraoperative navigation, and real-time decision support; the latter are of greatest relevance to the operating surgeon and are emphasized accordingly.

### 1.2 Thoracic imaging and radiology

Recent narrative and systematic reviews have reported improvements in diagnostic accuracy and intraoperative decision-making using a wide range of AI applications in thoracic surgery. These include chest CT interpretation, three-dimensional (3D) reconstruction for surgical planning, and robotic-assisted thoracoscopic surgery (RATS) (3, 4).

Multiple studies have shown that AI algorithms can achieve radiologist-level accuracy in detecting diverse thoracic pathologies (5, 6). Hendrix et al. (2023) reported sensitivities of 94%–97% for detecting both benign and malignant pulmonary nodules in non-screening CT scans, comparable to expert radiologists (7).

AI applications extend beyond CT. In chest radiography, a study comparing two datasets, AI-HUB (with image- and pixel-level CT-referenced annotations) and the NIH ChestX-ray14 (with image-level labels only), demonstrated superior performance of the AI-HUB-trained model, with area under the curve (AUC) values up to 0.91, reflecting excellent diagnostic accuracy (8).

Deep-learning reconstruction has also been shown to reduce image noise in ultra-low-dose CT, enhancing the detection and measurement of sub-centimeter pulmonary nodules. A study using four deep-learning models trained on CT scans from West China Hospital confirmed these improvements (9).

Beyond CT and radiography, advanced MRI protocols such as Cine-MRI and T1TSE have demonstrated value in differentiating benign from malignant mediastinal masses, further supporting accurate preoperative planning (10).

AI models have further enhanced malignancy risk estimation for small nodules (<1 cm) detected on screening chest CT, supporting clinical decision-making for intermediate lesions (11). For example, in a cohort comparison, the deep-learning-assisted model C-Lung-RADS (Chinese Lung Nodules Reporting and Data System) outperformed Lung-RADS v2022 in malignancy risk stratification (12).

Systematic reviews also support these findings. One review showed that deep-learning–based computer-aided diagnosis (DL-CADx) models improved risk stratification over clinical models alone, achieving 14.5% higher sensitivity and 7.4% higher specificity (13).

Automated segmentation and quantification of thoracic structures have also been enabled by AI (14). In the ImaLife cohort study, AI-derived measurements on low-dose CT for vertebrae, aorta, and coronary artery calcium (CAC) demonstrated excellent reliability, with intraclass correlation coefficients >0.9 (15).

A systematic review by Gao et al. (2025) on deep learning for pulmonary nodule detection highlighted concerns about bias and integration challenges but affirmed AI's potential to optimize workflows, including triage, volumetry, and detection support (16).

Reflecting these advances, the European Society of Thoracic Imaging (ESTI) has emphasized the importance of strong data curation, performance transparency, regulatory compliance, and training of radiologists in AI technologies (17).

### 1.3 Diagnosis and risk stratification

Malignancy risk estimation in CT-detected pulmonary nodules using deep-learning algorithms is now widely studied and increasingly validated (13). Compared with specialist judgment and clinical risk models, AI-based computer-aided diagnosis (CADx) has demonstrated superior performance, with one systematic review reporting gains of +11.6% in sensitivity and +7%–14% in specificity (13).

In direct comparisons, a lung-cancer-prediction convolutional neural network (LCP-CNN) outperformed multiparametric statistical methods such as the Brock model and Lung-RADS, a standardized framework developed by the American College of Radiology for categorizing lung nodules detected during CT screening (18). LCP-CNN achieved AUCs of 0.92–0.93 across populations that included patients with emphysema and interstitial lung disease, surpassing both Brock and Lung-RADS thresholds (18).

Beyond static prediction, time-series approaches have shown further promise. A longitudinal multimodal model integrating CT features, developed by Aslani et al. (2022), achieved an AUC of 0.88, surpassing static evaluations and matching radiologists' performance in assessing indeterminate nodules (19).

Nonetheless, AI models are not without limitations. Reduced generalizability across institutions, elevated false-positive rates, and inconsistent adherence to reporting standards remain ongoing concerns (18, 20, 21).

To allow direct comparison across studies, the principal performance metrics reported above are summarized in Table 1.

**Table 1:** Representative AI studies in thoracic imaging, diagnosis, and risk prediction cited in this review.

Study	AI approach	Clinical task	Population/dataset	Key performance	Principal limitation
Hendrix et al., 2023 (7)	Deep learning	Detection of benign and malignant pulmonary nodules	Non-screening chest CT	Sensitivity 94%–97% (radiologist-level)	Non-screening cohort; external generalizability untested
Kim et al., 2024 (8)	Deep learning (pixel-level labels)	Nodule detection on chest radiography	AI-HUB vs NIH ChestX-ray14	AUC up to 0.91	Performance depends on annotation quality
Wang J et al., 2025 (9)	Deep-learning reconstruction	Sub-centimeter nodule detection and measurement	Ultra-low-dose chest CT (single centre)	Improved detection and measurement accuracy	Single-centre data
Zhang R et al., 2024 (11)	Deep learning	Malignancy risk of incidental sub-cm nodules	Screening chest CT	Supports decisions for intermediate lesions	Limited to very small nodules
Wang C et al., 2024 (12) (C-Lung-RADS)	Data-driven stratification	Malignancy risk stratification	Chinese CT cohort	Outperformed Lung-RADS v2022	Needs external/prospective validation
Wulaningsih et al., 2024 (13) (DL-CADx)	Deep-learning CADx (meta-analysis)	Malignancy risk prediction	Pooled CT cohorts	+11.6% sensitivity; +7%–14% specificity vs clinical models	Between-study heterogeneity
Piskorski et al., 2025 (18) (LCP-CNN)	Convolutional neural network	Malignancy risk stratification	Cohorts incl. emphysema and ILD	AUC 0.92–0.93 (> Brock, Lung-RADS)	False positives; reporting inconsistency
Aslani et al., 2022 (19)	Longitudinal multimodal (time-series)	Cancer prediction in indeterminate nodules	Serial CT	AUC 0.88 (matched radiologists)	Requires prior imaging
Wang S 2025 (36); Zhou 2024 (37)	Explainable ML (SHAP)/PBNN	Prediction of postoperative pulmonary complications	Thoracoscopic surgery cohorts	AUC 0.73–0.82 (up to 0.87)	Single-centre; needs prospective validation

Taken together, the strongest and most reproducible evidence supports AI for pulmonary nodule detection and malignancy risk stratification on CT, where multiple independent cohorts and a meta-analysis report radiologist-level or superior discrimination (7, 12, 13, 18); evidence for these tasks is now mature enough to inform triage and reporting workflows. By contrast, results for longitudinal risk modeling and for ultra-low-dose or radiography-based detection, while promising, rest on fewer, often single-centre datasets and require external validation before routine adoption (8, 9, 19). Across all tasks, the recurring limitations are reduced generalizability to populations and scanners not represented in training data, elevated false-positive rates, and inconsistent adherence to reporting standards (18, 20, 21), which temper the headline metrics and should be weighed when these tools are considered for clinical use.

### 1.4 Surgical planning & intraoperative navigation

In preoperative planning, AI and deep-learning systems have played a significant role. AI-assisted three-dimensional (3D) reconstruction from CT scans produces highly accurate anatomical models, enhancing preoperative planning by enabling spatial visualization of lesions and adjacent structures beyond the capabilities of traditional two-dimensional imaging (22, 23).

Reported accuracy for classifying segmental bronchi, arteries, and veins has reached 97%–98% with AI-assisted 3D reconstruction systems in selected, curated cohorts, and has been associated with reduced conversion from planned lobectomy and other thoracic procedures (21). These figures should, however, be interpreted with caution. Segmental pulmonary vascular anatomy is highly variable (24, 25), and reconstruction algorithms trained on more typical configurations may misclassify anomalous arteries or veins, particularly small or aberrant branches; such errors can mislead rather than assist the surgeon and, if uncritically trusted, may increase intraoperative risk. Accuracy reported under ideal conditions therefore does not guarantee equivalent performance in the anatomically complex cases where navigation support is most needed, and the surgeon's independent anatomical judgment remains essential (26).

In video-assisted thoracoscopic surgery (VATS), the integration of AI has improved port and trocar planning. A virtual-reality (VR)–assisted system demonstrated measurable reductions in trocar placement errors and the need for intraoperative adjustments (27).

The first AI-enhanced augmented-reality (AR) robotic lobectomy was reported by Sadeghi et al. (2024), in which interactive overlays facilitated anatomical identification, improving spatial orientation and surgical safety (28).

Similarly, AI-based pulmonary vessel segmentation pipelines have been validated, supporting surgical planning for segmentectomies (29). Reviews of AI-assisted displays in thoracic surgery further confirm improvements in anatomical understanding during both pre- and intraoperative workflows (30).

AI is also being adopted for real-time decision support and evaluation of surgical performance. A narrative review by Knudsen et al. (2023) described AI-enhanced robotic systems capable of force estimation, real-time margin detection, and automated responses to operative video, thereby enhancing intraoperative decision-making and surgical education (31). Surgeons using AR-guided robotic systems have reported improved visualization of tumor boundaries, more accurate identification of vascular structures, and shorter dissection times (28, 29).

It should be emphasized that these surgeon-facing applications—augmented-reality overlays, intraoperative tumour-boundary detection, and real-time margin assessment—are at an earlier evidentiary stage than the diagnostic tasks discussed above, resting largely on single-institution feasibility reports and small case series rather than comparative or randomized data (28, 31). Their promise for operative practice is considerable, but the current level of evidence remains preliminary.

### 1.5 Integrating AI across the thoracic surgical pathway

While specific applications of AI in imaging, risk stratification, and surgical planning have been outlined, its broader integration across the entire thoracic surgical pathway is equally significant. AI technologies now extend beyond diagnosis and intraoperative support to encompass perioperative and postoperative management, offering opportunities to personalize care and improve outcomes (32, 33).

AI has also demonstrated value in thoracic oncology, and because lung cancer is the central indication of thoracic surgery its role there warrants specific attention. In non-small cell lung cancer (NSCLC), AI has been applied to histological and molecular characterization, prediction of lymph node metastasis, radiomic phenotyping of tumours, and estimation of treatment response and prognosis, in addition to supporting efficient extraction of electronic medical record (EMR) data; collectively these applications may refine patient selection, extent-of-resection decisions, and surveillance strategies (33–35). As with the imaging tasks above, most of this evidence derives from retrospective single-centre cohorts, and prospective validation is needed before these models guide operative decisions.

In perioperative care, machine-learning models can predict postoperative pulmonary complications (PPCs) with high accuracy, enabling proactive interventions and tailored monitoring strategies. Explainable ML models have achieved AUCs of 0.73–0.82 in thoracoscopic surgery cohorts, while other studies report AUCs up to 0.87 for PPC prediction, demonstrating feasibility for early risk stratification and resource optimization (36, 37).

These models typically draw on a defined set of clinically interpretable variables—baseline pulmonary function (e.g., FEV1 and diffusing capacity), age, smoking history, comorbidity burden, the extent and approach of resection, and intraoperative parameters—rather than operating as opaque predictors. Making the contribution of individual variables explicit is important for clinical trust: the thoracoscopic-cohort model cited above uses SHapley Additive exPlanations (SHAP) to show which factors drive an individual patient's predicted risk (36). Such explainable-AI approaches allow the surgeon to interrogate and, where appropriate, override a prediction, and are increasingly regarded as a prerequisite for integrating risk models into perioperative decision-making.

Pilot studies combining wearable sensors with ML algorithms have further shown the ability to detect adverse postoperative events up to two days before symptom onset, enhancing recovery pathways and enabling earlier clinical intervention (38).

Beyond complication prediction, AI-driven natural language processing (NLP) can extract and analyze unstructured electronic medical record data, supporting research, quality improvement, and real-time clinical decision-making (1). Emerging concepts such as explainable AI (XAI) aim to improve transparency and clinician trust, while digital twin models—virtual patient-specific simulations—are being developed to optimize surgical planning, training, and outcomes (39–41).

Realistic clinical translation also requires attention to health economics and accountability, which have received limited consideration to date. AI deployment carries the costs of software acquisition, integration with existing imaging and record systems, validation, and ongoing maintenance and monitoring, and the cost-effectiveness of most thoracic applications has not been formally established. Questions of liability are equally unresolved: when an adverse event—such as major haemorrhage following an erroneous AI-generated vascular reconstruction or navigation cue—occurs, responsibility may be distributed among the operating surgeon, the software developer, the device manufacturer, and the institution, and current legal frameworks provide little guidance. These considerations are developed further in Section 4.2.

In summary, the clinical maturity of AI varies markedly across the thoracic pathway. Nodule detection and malignancy risk stratification on CT are supported by the strongest evidence and are closest to routine adoption; automated segmentation and perioperative risk prediction are feasible and promising but await prospective validation; and the intraoperative, surgeon-facing applications—augmented-reality navigation and real-time margin or boundary assessment—remain at the proof-of-concept stage despite their high potential relevance to operative practice. Recognizing where each application sits on this spectrum is essential to deploying AI responsibly rather than uniformly.

## 2 Development of robotic surgery in thoracic surgery

Robotic surgery, first introduced in the late 1980s, has steadily expanded across multiple surgical specialties, with thoracic surgery becoming a notable adopter (42). The field is undergoing a major transition from video-assisted thoracoscopic surgery (VATS) to robotic-assisted thoracic surgery (RATS), and RATS is increasingly adopted for minimally invasive procedures, although—as discussed below—its position relative to VATS is not yet established by level-1 evidence (42, 43). Robotic systems provide superior 3D visualization, magnified views, and articulated instruments that replicate the dexterity of the human wrist, thereby improving precision and control during complex operations (42, 43). These advantages have translated into improved outcomes, including fewer complications, reduced postoperative pain, and faster recovery compared with open surgery and, in some cases, even VATS (43–45).

The benefits of robotic platforms extend across a wide range of thoracic procedures, including lung resections, mediastinal surgery, and esophageal operations (43). Robotic segmentectomy for lung cancer has shown promising results in prospective analyses (46). Multicenter studies indicate that RATS can reduce perioperative complications while achieving long-term survival outcomes comparable to posterolateral thoracotomy in patients with clinical N2 stage NSCLC (45). The versatility of robotic platforms allows them to be applied across both straightforward resections and complex cases involving lymph node dissection (43, 45).

Robotic approaches have also been applied specifically to esophageal resection in the form of robot-assisted minimally invasive esophagectomy (RAMIE). Compared with conventional minimally invasive esophagectomy, RAMIE has been associated with more extensive mediastinal lymphadenectomy—particularly along the recurrent laryngeal nerves—and with favourable short-term outcomes, while the wristed instrumentation facilitates the technically demanding intrathoracic anastomosis; these advantages are balanced by a defined learning curve (47).

Recent clinical experience has also shown the feasibility of contralateral robotic-assisted anatomical resections for patients with synchronous or metachronous lung cancer, supporting the extension of RATS indications to complex bilateral disease (48).

Recent retrospective evidence also supports the safety and feasibility of RATS for large thymomas, demonstrating comparable oncologic outcomes to open surgery while preserving the benefits of minimally invasive access (49). Robotic approaches have also been successfully applied to posterior mediastinal neurogenic tumors, where enhanced visualization and precise dissection facilitate safe resection in anatomically challenging locations (50).

Comparative studies further highlight the advantages of RATS. Propensity score–matched analyses comparing RATS and VATS for NSCLC have been conducted across diverse patient populations, including older patients (51, 52), younger patients (53), patients with varying BMI ranges (54), those with coronary artery disease (55), and those undergoing neoadjuvant therapy (56, 57) or chemotherapy (58). These analyses underscore the growing evidence base supporting RATS across multiple clinical contexts.

A balanced comparison must also consider uniportal VATS, which is now widely practised and is not captured by comparisons of RATS with conventional multiportal VATS. Uniportal VATS performs lobectomy and segmentectomy through a single 3–4 cm incision, whereas most RATS procedures still require three to four ports. In a recent systematic review and meta-analysis, the single-incision approach was associated with a clinically meaningful reduction in early postoperative pain — a standardised mean difference of approximately −0.98 at 24 h, equating to roughly 2.5 points on a 10-point scale — and with lower opioid consumption than multiportal VATS (59). RATS, in turn, offers wristed articulation, tremor filtration, and a stable magnified three-dimensional view that can facilitate complex mediastinal and nodal dissection. The two approaches are therefore best regarded as complementary; the choice depends on tumour and patient factors, institutional resources, and surgeon expertise rather than on a uniform superiority of either platform.

Despite these benefits, important limitations remain. Robotic surgery requires a steep learning curve, necessitating extensive training and practice (60–62). The financial burden is substantial and extends well beyond the initial acquisition of the system to specialized consumables and instruments, service contracts, and maintenance; these costs raise the average per-patient expenditure and constrain access, a barrier that is particularly acute in developing and resource-limited settings, and formal cost-effectiveness data remain limited (60).

The evidence base itself also warrants caution. Although numerous propensity-score-matched analyses report outcomes comparable or superior to VATS across varied subgroups (44, 51–58), no large randomized controlled trial has directly compared RATS and VATS for major lung resection with respect to long-term oncological outcomes, and the available randomized data compare robotic surgery chiefly with open thoracotomy rather than with VATS (45). Propensity-score matching reduces measured confounding but cannot eliminate unmeasured confounders—most notably surgeon experience and centre volume—so current comparative claims should be regarded as supportive rather than definitive (42, 43).

A further clinically important limitation of current systems is the absence of haptic (tactile) feedback. Without a direct sense of tissue tension, the surgeon relies on visual cues to gauge force, which can lead to inadvertent excessive traction or shear, tissue tearing, or injury to vessels during dissection and retraction. This risk is mitigated in part by high-fidelity three-dimensional vision and by growing experience, and it is beginning to be addressed at the hardware level: the recently introduced da Vinci 5 platform incorporates force-feedback capability into the clinical system, with pre-clinical data reporting reductions in the force applied to tissue of up to 43%, irrespective of surgeon experience (63). This represents a tangible, already-deployed advance rather than a speculative future feature.

Nodal assessment illustrates both the promise and the current evidentiary limits of RATS, and here the quantitative data are genuinely mixed. In one propensity-matched series, VATS dissected significantly more nodal stations than RATS (5.9 ± 1.5 versus 4.8 ± 2.2) (44), whereas meta-analyses of comparative studies have reported the opposite, with a significantly higher number of lymph nodes dissected during RATS (64). Most series nonetheless report comparable overall nodal yield and similar upstaging rates, so on current evidence the two approaches are best regarded as achieving broadly equivalent nodal assessment (44, 64).

In addition, regulatory changes such as the EU Medical Device Regulation (MDR) and evolving FDA guidance for AI/ML-enabled devices have increased requirements for clinical validation, post-market surveillance, and algorithm transparency, raising development costs and creating barriers for smaller companies (65, 66).

Efforts to address these challenges include standardizing robotic surgery training, with expert consensus statements providing frameworks for national curricula (67, 68). Manufacturers are also developing more affordable robotic alternatives and incorporating clinician feedback into design to improve usability and reduce the learning curve (60). A leading future priority remains the continued restoration of haptic feedback, which would further enhance surgical precision. The evolution of robotic thoracic surgery continues to promise increasingly precise, safe, and effective interventions.

In summary, RATS is an established, safe, and increasingly adopted approach that reproduces and in some respects extends the benefits of minimally invasive thoracic surgery, with its clearest demonstrated value in complex mediastinal resections and in reproducing VATS-level perioperative outcomes across diverse patient groups. Its superiority over contemporary VATS—including uniportal VATS—for oncological endpoints remains unproven in the absence of randomized data, and cost, access, and the lack of haptic feedback remain genuine limitations. RATS is therefore best described as a mature and expanding option rather than an unequivocal new standard.

## 3 Telesurgery in thoracic surgery

Telesurgery represents a groundbreaking advancement in surgical practice, enabling surgeons to operate on patients located at great distances (69). This innovative approach has the potential to overcome geographical barriers and optimize the allocation of specialized surgical expertise worldwide (70). The rapid evolution of 5G technology has been a critical enabler, providing the ultra-low latency and high data-transfer speeds essential for real-time telesurgical operations (70–72). This infrastructure allows the seamless transmission of control signals, high-definition images, and audio, enabling surgeons to perform complex procedures with unprecedented precision from a distance (70).

Thoracic applications have already demonstrated feasibility. In December 2024, a successful ultra-remote robot-assisted right upper lobectomy with lymph node dissection was performed between a surgeon in Shanghai and a patient in Kashi, separated by more than 5,000 km, using a dedicated 5G network (70). The average latency was ∼100 ms, well below the generally accepted safety threshold of 200–300 ms for telesurgical control (73). The patient experienced an uneventful recovery, with no connection interruptions.

This landmark case should be understood as a proof of concept achieved under near-ideal, purpose-built conditions—a dedicated point-to-point 5G link, redundant infrastructure, and an experienced bedside team—rather than as evidence of routine clinical deployability. A systematic review confirmed that human telesurgery cases remain rare across surgical specialties, reinforcing this distinction and underscoring the pioneering nature of thoracic experiments (73).

Notable achievements in telemedicine and telesurgery highlight the feasibility and safety of this approach. Telesurgery expands access to specialized care, particularly benefiting patients in remote or underserved regions who may otherwise lack access to expert surgeons (70, 74). Dual- and multi-console capabilities, enhanced by 5G connectivity, further enable global collaboration, allowing multiple surgeons to participate in or supervise procedures simultaneously. This capacity facilitates real-time consultation and intervention, especially useful in complex or emergency cases (70, 75). AI integration is also being explored to mitigate potential delays in telesurgery and telementoring, further enhancing its scope (76). Systematic reviews have outlined the current state and future directions of 5G-enabled telesurgery (70, 77–79).

Despite its promise, widespread adoption faces significant challenges. Key concerns include network reliability, cybersecurity vulnerabilities, and the need for standardized global protocols to ensure safety in remote surgical practice (70, 76, 80). Network latency and bandwidth remain critical factors that require continuous optimization to maintain precision and patient safety (81).

From the operating surgeon's perspective, the foremost clinical risk of telesurgery is the management of sudden, catastrophic intraoperative complications. A tear of a pulmonary artery branch during hilar dissection—for example, during a right upper lobectomy—can produce massive haemorrhage within minutes, a situation in which the remotely located console surgeon cannot physically intervene. Safe telesurgical practice therefore depends on a fully capable bedside surgical team able to obtain immediate haemostasis and, if necessary, convert to open thoracotomy without delay, together with explicit emergency-conversion protocols agreed in advance. The physical separation of surgeon and patient introduces an irreducible rescue latency, and this consideration—rather than average network latency alone—may be the principal determinant of which procedures and patients are appropriate for remote surgery.

These considerations argue for careful patient and procedure selection, which the current literature has largely left implicit. Although the reported right upper lobectomy was successful, a procedure carrying a high risk of major haemorrhage is not an ideal model for routine telesurgery. Lower-risk procedures—such as resection of benign mediastinal lesions, thoracic sympathectomy for hyperhidrosis, and peripheral wedge resection—are better suited to early clinical deployment, and a formal risk-stratification framework that weighs bleeding risk, anatomical complexity, and the capability of the local team should guide case selection as the field matures.

Regulatory and medico-legal barriers are also more substantial than is often acknowledged, and they apply even to practice within a single country. When a surgeon in one jurisdiction operates on a patient in another—as in a procedure spanning distant regions of the same country—questions of medical licensing and registration, prescribing authority, and the designation of legal responsibility are not straightforward. A workable framework must specify how liability is allocated among the console surgeon, the bedside team, the platform manufacturer, and the network provider when an adverse event occurs, and this will require deliberate policy development rather than the extension of existing single-site rules. Ethical considerations are equally important, including patient autonomy, informed consent in a remote context, and robust data-privacy frameworks to safeguard sensitive patient information during transmission and storage (76).

Finally, the framing of telesurgery as a solution to inequitable access deserves scrutiny. Successful remote surgery requires not only ultra-reliable, low-latency connectivity but also a compatible robotic platform on site, a skilled bedside surgical assistant, an anaesthetist, and a trained perioperative team—precisely the infrastructure and personnel that are typically absent in the underserved or remote settings that telesurgery is proposed to serve. In its current form, telesurgery is therefore unlikely to function as a stand-alone remedy for access disparities. Its more realistic and immediately valuable near-term role is as a platform for inter-institutional collaboration, proctoring, and surgical telementoring between well-resourced, high-volume centres, through which expertise can be shared and disseminated even where fully independent remote operating is not yet feasible (70, 82).

In summary, telesurgery in thoracic surgery has been shown to be technically feasible in a small number of pioneering cases performed under dedicated conditions, but it remains firmly at the proof-of-concept stage. The principal unresolved barriers are not only technical—network redundancy and real-world latency variability—but also clinical (intraoperative crisis management and patient selection) and medico-legal (cross-jurisdiction licensing and liability). Its most credible near-term contribution is collaborative and educational rather than as a scalable solution to surgical access.

## 4 Challenges, limitations, ethical considerations, and future directions in emerging technologies

The rapid integration of artificial intelligence (AI), robotics, and telesurgery into thoracic surgery brings unprecedented opportunities, but also substantial challenges and ethical dilemmas that must be addressed before widespread, equitable adoption. These concerns span technical, clinical, ethical, and societal domains.

### 4.1 Technical and clinical limitations

AI models often struggle with generalizability when applied across diverse populations. Systems trained on homogeneous or limited datasets may perform poorly in other contexts, risking the exacerbation of healthcare disparities (32). Similarly, AI algorithms may learn “shortcuts,” such as associating hospital-specific imaging artifacts with disease, leading to inflated performance metrics but reduced clinical reliability (83). High false-positive rates and inconsistent adherence to reporting standards remain ongoing concerns (18, 20, 21).

Beyond these general concerns, thoracic surgery poses field-specific obstacles to AI generalizability. Pulmonary vascular and bronchial anatomy is highly variable—segmental arterial and venous branching patterns deviate from the textbook configuration in a substantial proportion of patients—so models and reconstructions trained on more typical anatomy carry a real risk of misclassification precisely in the anomalous cases where accurate guidance matters most (26). Three-dimensional CT studies of patients undergoing VATS lobectomy document substantial inter-individual variation in the pulmonary arterial tree (24), and variant venous patterns such as anomalous middle-lobe drainage or a common trunk of the left pulmonary veins each occur in roughly 10%–25% of patients (25).

A further, distinctively thoracic limitation concerns intraoperative navigation. Preoperative three-dimensional models and augmented-reality overlays are derived from full-inspiration CT, yet thoracic surgery is conducted under one-lung ventilation with the operative lung deflated. The resulting collapse produces substantial dynamic deformation and spatial mismatch between the preoperative model and the intraoperative anatomy, degrading the accuracy of navigation cues and complicating their use to guide robotic manipulation. Reliable compensation for this deformation remains an unsolved problem and is a key obstacle to trustworthy intraoperative AI guidance in the chest.

In robotic surgery, challenges include the steep learning curve, high procedural complexity, and the absence of haptic feedback, which can impair surgical precision (30, 42). Telesurgery, while promising, depends on robust 5G networks; latency, bandwidth fluctuations, and cybersecurity vulnerabilities remain critical limitations (70, 76, 80).

### 4.2 Ethical and legal accountability

The integration of advanced technologies necessitates clear ethical and legal frameworks. A core ethical challenge is algorithmic bias, where AI models trained on non-diverse data may introduce systemic disparities in patient care (84). This requires continuous auditing of training data and model performance across varied demographics to ensure fairness and generalizability (17). Furthermore, the principle of informed consent must be updated to clearly communicate the role and limitations of AI-driven decision support systems to patients (85).

Legally, the question of liability remains complex, particularly in the event of an adverse outcome during an AI-assisted or robotic procedure. Determining responsibility among the surgeon, AI developer, device manufacturer, and hospital system requires new legal precedents and regulatory clarity (86). For telesurgery, the legal jurisdiction and medical licensing across international borders pose additional challenges, demanding standardized international protocols for credentialing and malpractice coverage (87).

### 4.3 Future directions in responsible innovation

Addressing these challenges requires genuine multidisciplinary collaboration. The teams that design and deliver these technologies should include not only surgeons, engineers, ethicists, policymakers, and patient representatives but also, specifically, anaesthesiologists—whose management of one-lung ventilation and of intraoperative crises is central to the safety of both robotic and remote thoracic surgery—and network and communication engineers, whose expertise in latency, redundancy, and data security is indispensable to telesurgical deployment. Initiatives should focus on creating diverse and representative datasets, developing explainable AI (XAI) to increase transparency, restoring haptic feedback in robotic systems, and standardizing global telesurgical protocols.

Looking further ahead, the integration of AI into the robotic platform itself—advancing from decision support toward semi-autonomous execution of defined operative steps under surgeon supervision—is a plausible long-term direction, but one that will demand rigorous validation and clear lines of accountability before clinical use (42, 88). Comparable caution applies to the concept of the digital twin. A faithful patient-specific virtual replica of the lung is far more difficult to construct than a model of rigid bone or a solid abdominal organ, because the lung is a dynamic organ defined by ventilation, perfusion, deformable soft tissue, and continuously changing compliance; capturing this behaviour in a data model that remains accurate intraoperatively is a formidable and as-yet-unrealized challenge, and claims for digital-twin-guided thoracic surgery should be read with this in mind (39–41). Ethical frameworks must evolve in parallel with technological development, ensuring that innovation is balanced by safety, accountability, and equitable access.

## 5 Convergence toward integrated digital surgical ecosystems

Considered individually, artificial intelligence, robotics, and telesurgery each address a distinct part of the surgical pathway. Arguably the most consequential near-term development, however, lies not in any one of these technologies in isolation but in their convergence into integrated digital surgical ecosystems, in which AI-driven intraoperative guidance is delivered through the robotic platform, real-time telementoring connects operating surgeons across sites, and a shared data infrastructure links preoperative planning, intraoperative execution, and postoperative outcomes. In such a system the three domains reinforce one another: AI supplies decision support and navigation, the robotic platform provides the precise actuation through which that support is expressed, and connectivity enables remote collaboration, proctoring, and continuous learning from pooled operative data.

Commercial and academic platforms are increasingly designed with this integration in mind. Established surgical ecosystems, such as those built around the Intuitive Surgical platforms and the Medtronic Hugo system, together with emerging open-architecture robotic platforms are moving toward embedded analytics, networked connectivity, and interoperable data capture rather than functioning as standalone instruments. Realizing this vision responsibly will require the same safeguards emphasized throughout this review; representative data, transparent and explainable models, robust cybersecurity and data governance, clear allocation of accountability, and equitable access, applied now at the level of the integrated system rather than the individual tool. Understood in this way, the trajectory of thoracic surgical technology is less a set of parallel advances than a convergence toward a connected, data-driven operative environment.

## 6 Conclusions and future directions

The convergence of AI, robotics, and telesurgery is reshaping thoracic surgery toward greater precision, personalization, and minimally invasive care. AI enhances diagnosis and planning, RATS improves operative dexterity, and telesurgery extends expert reach; yet, as this review has emphasized, the clinical maturity of these technologies varies widely, and their benefits must be weighed against unresolved limitations in evidence, cost, safety, and equity.

The next phase of development will focus on integration and disciplined refinement rather than on capability alone. This includes restoring haptic feedback in robotic systems, cautiously exploring semi-autonomous functions under rigorous validation, compensating for the distinctive intraoperative challenges of the chest, and establishing robust, internationally harmonized ethical and legal frameworks. Progress should be judged not only by what these technologies make possible, but by what is proven, adopted in routine practice, and equitably accessible.

Ultimately, the future of thoracic surgery depends on a globally collaborative model that ensures these powerful technologies are leveraged not only for clinical excellence but also for equitable access to advanced care worldwide.
